# Systematic Evaluation of PKH Labelling on Extracellular Vesicle Size by Nanoparticle Tracking Analysis

**DOI:** 10.1038/s41598-020-66434-7

**Published:** 2020-06-12

**Authors:** Mehdi Dehghani, Shannon M. Gulvin, Jonathan Flax, Thomas R. Gaborski

**Affiliations:** 1 Department of Microsystems Engineering, Rochester Institute of Technology, Rochester, NY United States; 2 Department of Biomedical Engineering, Rochester Institute of Technology, Rochester, NY United States; 3 Department of Urology, University of Rochester Medical Center, Rochester, NY United States; 4 Department of Biomedical Engineering, University of Rochester, Rochester, NY United States

**Keywords:** Wide-field fluorescence microscopy, Nanobiotechnology, Cellular imaging, Membrane trafficking

## Abstract

Extracellular vesicles (EVs) are membrane vesicles secreted by cells and can modulate biological activities by transferring their content following uptake into recipient cells. Labelling of EVs is a commonly used technique for understanding their cellular targeting and biodistribution. A reliable fluorescent technique needs to preserve the size of EVs since changes in size may alter their uptake and biodistribution. Lipophilic fluorescent dye molecules such as the PKH family have been widely used for EV labelling. Here, the effect of PKH labelling on the size of EVs was systematically evaluated using nanoparticle tracking analysis (NTA), which is a widely used technique for determining the size and concentration of nanoparticles. NTA analysis showed a size increase in all the PKH labelling conditions tested. As opposed to lipophilic dye molecules, no significant shift in the size of labelled EVs was detected with luminal binding dye molecules such as 5-(and-6)-carboxyfluorescein diacetate succinimidyl ester (CFDA-SE, hereinafter CFSE). This finding suggests that PKH labelling may not be a reliable technique for the tracking of EVs.

## Introduction

EVs are small membrane bound vesicles (50–1000 nm in diameter) secreted by all cell types examined and can be found in almost all biofluids including blood, breast milk, urine and saliva as well as in cell culture media^1,2^. EVs mediate cell-cell communication by exchanging proteins, DNA, RNA and lipids between donor and recipient cells and activating signaling pathways in target cells via receptor ligand interaction^3,4^. They have been shown to play a role in regulating both physiological and pathological processes including immune regulation and cancer^5^.

Studies that examine EVs uptake into target cells and *in vivo* biodistribution have utilized a range of labelling and tracking approaches to follow EVs fate^6^. The most common technique for studying EVs biodistribution and target cell interaction involves labelling of EVs with fluorescent dye molecules. Many strategies have been developed for labelling EVs such as staining their membranes using fluorescent lipid membrane dye molecules such as PKH26^7,8^, PKH67^9,10^, DiI^11^, and DiD^12^. The PKH family has been widely used in the lipophilic class as they have a highly fluorescent polar head group and long aliphatic hydrocarbon tails which readily intercalate into any lipid structure leading to long-term dye retention and stable fluorescence^12,13^. Using fluorescent labelling, EVs secreted by cells into the extracellular environment have been found to be internalized through different routes and mechanisms including fusion with the plasma membrane and a range of endocytic pathways such as receptor-mediated endocytosis, phagocytosis, lipid raft-dependent endocytosis and micropinocytosis^14–16^. Furthermore, the internalization of EVs has been shown with a wide range of recipient cells such as dendritic cells^9^, macrophages^17^, dermal fibroblast^18^, endothelial and myocardial cells^10^.

Previous studies have shown that the fate of nanoparticles can be affected by the size, shape, surface chemistry and hydrophobicity of nanoparticles^19–22^. In particular, the size-dependent uptake of nanoparticles composed of inorganic materials including those made of polystyrene^23^ and silica^24^ has been conclusively studied. Lower cellular uptake of nanoparticles has been consistently observed with increasing nanoparticle size, possibly due to the increased energy required to take up the larger nanoparticles^24–27^. Additionally, size impacts the biodistribution of nanoparticles *in vivo*. For instance, more rapid accumulation of larger nanoparticles was observed to take place in liver and spleen^28^. Large nanoparticles also tend to exhibit shorter circulation half-life, which may be due to the activation of the complement system and quick removal of large nanoparticles from blood^29^. Therefore, size of nanoparticles plays an important role in uptake, biodistribution and circulation half-life of nanoparticles.

Since previous studies have shown that cells preferentially uptake smaller EVs^30^, altering the size of EVs may also affect their uptake into target cells. Therefore, a reliable fluorescent dye must preserve native properties of EVs, such as size, after labelling. Despite the importance of preserving the size of EVs for uptake and biodistribution studies, the effect of the widely used PKH dye on the size of EVs has never been systematically characterized. The objective of this study was to systematically evaluate the effect of PKH labelling on the size of EVs using nanoparticle tracking analysis (NTA). NTA is a technique for measuring the size and concentration of nanoparticles in real time based on tracking the light scattered from suspended nanoparticles undergoing Brownian motion^25,31,32^.

In the present work, the ratio of PKH dye molecules to EVs was systematically varied and the particles’ size distribution were measured using NTA. In all the labelling conditions tested by NTA, EVs size mode increased after labelling. The observed size shift may trigger abnormalities in their tissue distribution and cellular uptake in both *in vivo* and *in vitro* studies. In contrast to PKH labelling, CFSE-labelled EVs showed similar size distribution as unlabelled EVs indicating that CFSE preserves the size of EVs.

## Results

**Figure 1:**
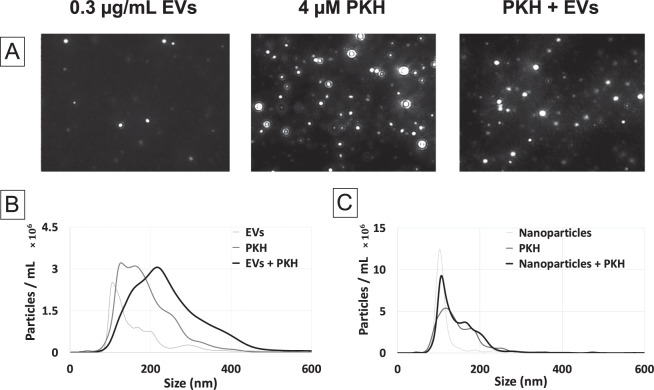
Size characterization of PKH labelling of EVs by Nanoparticle Tracking Analysis (NTA). (**A**) NTA video frames of 0.3 µg/mL EVs only as control (EVs), 4 µM PKH only as control (PKH), and PKH-labelled EVs (PKH + EVs) in diluent C. (**B**) Size distribution of EVs only control (EVs), PKH only control (PKH), and PKH-labelled EVs (PKH + EVs) samples in diluent C (n = 7). (**C**) Size distribution of PS nanoparticles only control, PKH only control, and PS nanoparticles in PKH in diluent C (n = 7).

In order to investigate the effect of PKH labelling on the size of EVs, the particles’ size distribution was assessed by NTA. A heterogenous population of nanoparticles with a typical size range of small EVs (80–300 nm) was found in the EVs only control. Moreover, the PKH only control (without EVs) contained PKH nanoparticles, possibly micelles and aggregation, with a polydisperse particles’ distribution in the size range of 80–400 nm (Fig. 1A,B), which is in agreement with previously reported results^33,34^. Furthermore, after labelling EVs with PKH, larger particles than those found in either the EVs or PKH only controls were detected by NTA (Fig. 1A,B), suggesting a size shift towards larger particles after PKH labelling. In order to confirm that PKH is the reason for the observed size shift after labelling EVs, the interaction of 100 nm polystyrene (PS) nanoparticles with PKH was studied as a control experiment. In contrast to EVs, PKH should not interact with PS nanoparticles and as expected, no size shift towards larger particles was observed by NTA when 100 nm PS nanoparticles were added to PKH (Fig. 1C). Therefore, labelling EVs with PKH caused a size shift towards larger sizes.

Possible mechanisms causing the observed size increase are the aggregation/fusion of PKH nanoparticles with EVs or the intercalation of PKH molecules into EVs membranes, both of which would result in the formation of larger species. This suggests the possibility of minimizing the size increase by reducing the PKH concentration, while maintaining detectable fluorescent signal from PKH labelled EVs. To assess this possibility, the fluorescent detection range of PKH-labelled EVs samples was first determined. PKH to EVs ratios were adjusted by changing the concentration of PKH while holding the EVs concentration constant. The fluorescent level of these samples along with that of the PKH and EVs only, as well as background only control groups were visualized using fluorescent microscopy and quantified by sampling the cross-sectional fluorescent intensity of the captured images (line scan).

**Figure 2:**
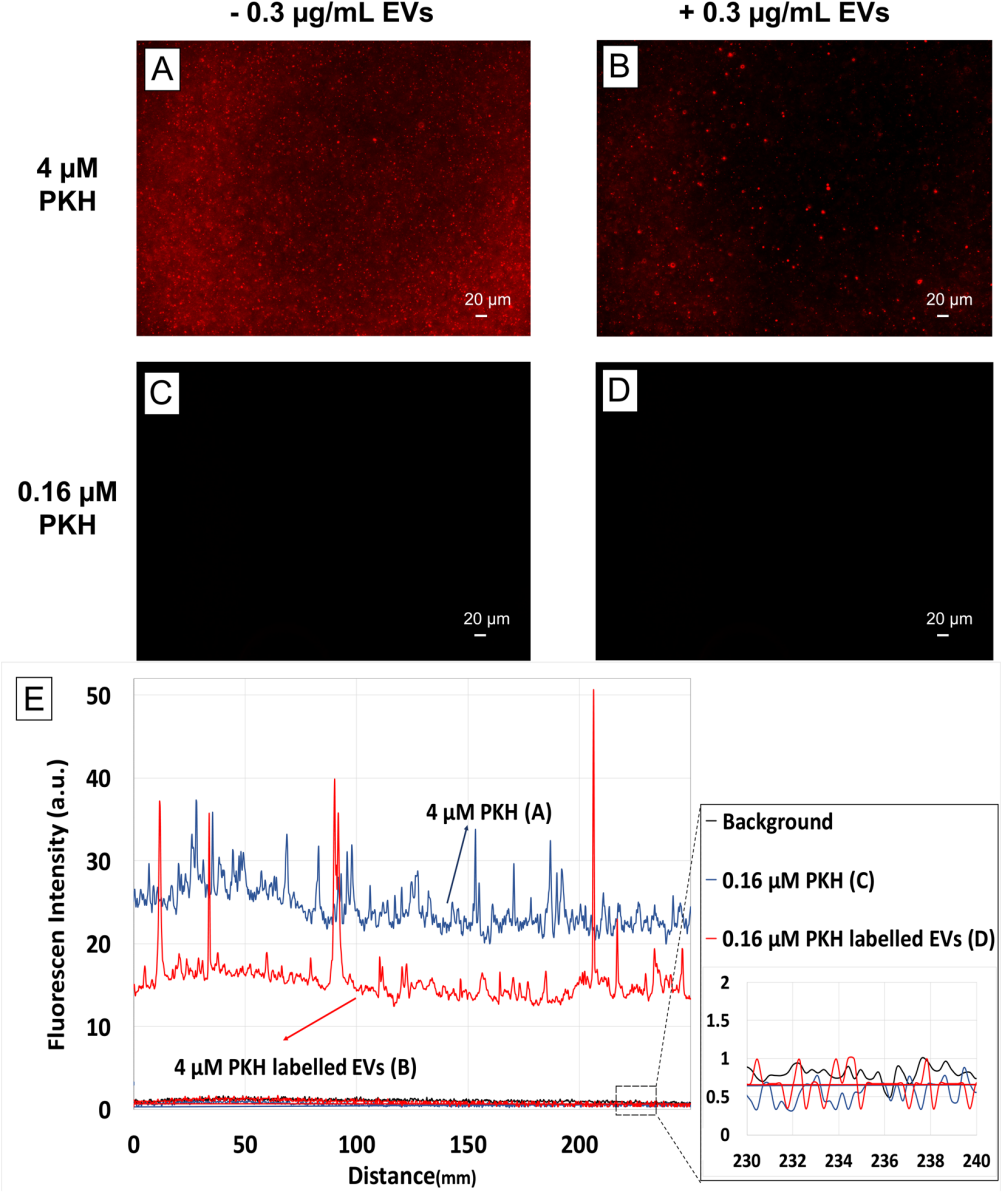
Determination of the fluorescent detection range. Fluorescent images of (**A**) 4 µM PKH only control, (**B**) 4 µM PKH-labelled EVs (1.5 µg/mL), (**C**) 0.16 µM PKH only control,(**D**) 0.16 µM PKH-labelled EVs (0.3 µg/mL) in diluent C. (**E**) Representative line scan analysis of fluorescent images (**A**–**D**). Inset shows the line scan analysis of background, 0.16 µM PKH only control and 0.16 µM PKH-labelled EVs (0.3 µg/mL) in diluent C.

Initially, the same concentrations of PKH and EVs were used as in Fig. 1. Weakly fluorescent features consistent with the presence of PKH nanoparticles in the PKH only control were observed by fluorescent imaging (Fig. 2A). In comparison to the PKH only control, several brighter features were observed in PKH labelled EVs which are likely the larger particles formed after PKH labelling of EVs (Fig. 2B). The line scan taken from the PKH-labelled EVs showed higher fluorescent intensity compared to the background signal (Fig. 2E). Line scan analysis of the PKH-labelled EVs revealed a reduction in the baseline signal when compared to the PKH only control; possibly as the result of floating PKH dye molecules interacting with EVs (Fig. 2E). Furthermore, intensity spikes found in the PKH-labelled EVs further support the presence of larger labelled EVs (Fig. 2E). In contrast, a 25-fold reduction of the PKH concentration (0.16 µM) led to a decrease in the fluorescent intensity that reached background level. This PKH reduction also caused the fluorescently bright features, observed at higher concentrations of PKH, to fall below the detectable limit (Fig. 2C–E).

**Figure 3:**
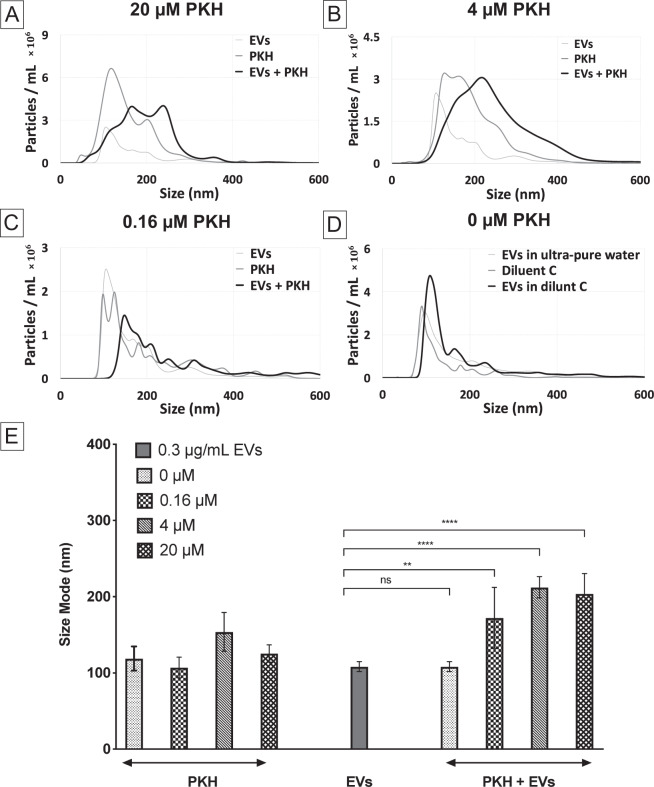
Effect of PKH concentration on the size distribution of particles in PKH-labelled EVs by nanoparticle tracking analysis (NTA). (**A**–**D**) Size distribution of PKH-labelled EVs with different PKH concentrations (20, 4, 0.16 and 0 µM respectively) with 0.3 µg/mL of EVs in diluent C (n = 7). (**E**) Particle size modes for EVs only control, PKH only controls, and PKH-labelled EVs (error bars represent the 95% confidence interval of the mean) in diluent C (n = 7, *p* value <0.05).

After determining the fluorescent detection range of PKH-labelled EVs, the effect of PKH concentration on the particles’ size distribution was explored by NTA. The concentration of PKH was systematically varied while holding EVs concentration constant. Representative examples of the particles’ size distribution measured by NTA for different concentrations of PKH can be seen in (Fig. 3A–D). For all concentrations of PKH, NTA analysis of particles’ size distributions showed that labelling with PKH caused the formation of larger species relative to EVs and PKH only controls. Quantitative determination of NTA results was done by comparing the modes of the nanoparticle sizes (Fig. 3E). Consistent with size distribution results, a shift in the modes towards larger particles was observed in all PKH-labelled EVs (PKH + EVs) compared to the EVs only control. Furthermore, no size shift was observed when the suspension buffer (diluent C) was added to EVs in the absence of PKH confirming that PKH is the cause for the size shift observed (Fig. 3D). Therefore, labelling EVs with different concentrations of PKH, even for the PKH concentration below the fluorescent detection limit, showed a size distribution shift indicative of the generation of larger species. This finding suggests that minimizing the formation of larger species by reducing the concentration of PKH used for labelling EVs while maintaining the fluorescent detectability may not be feasible.

**Figure 4:**
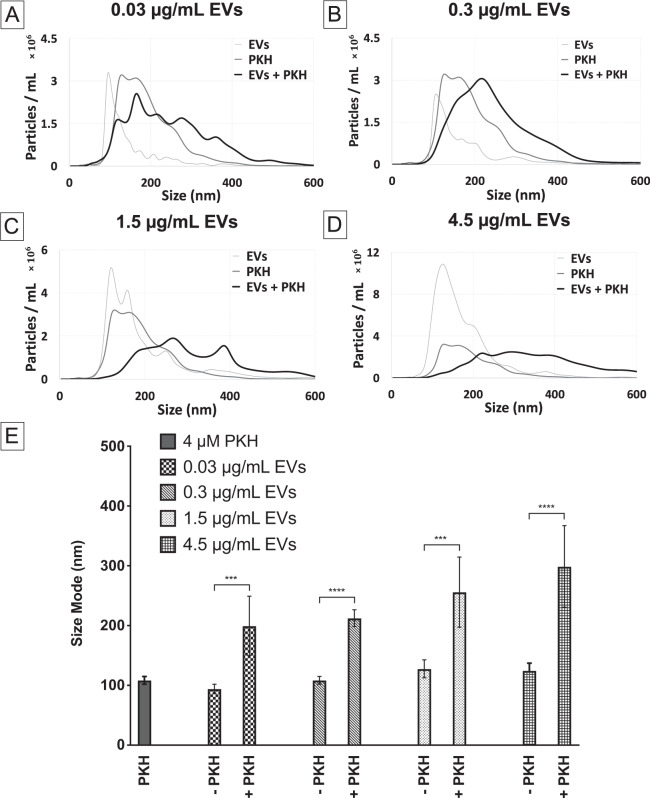
Effect of EVs concentration on the size distribution of particles in PKH-labelled EVs evaluated by nanoparticle tracking analysis (NTA). (**A**–**D**) Size distribution of PKH-labelled EVs with different EVs concentrations (0.03, 0.3, 1.5, 4.5 µg/mL respectively) with 4 µM of PKH (n = 7) in diluent C. (**E**) Particle Size modes of EVs only control, PKH only controls, and PKH-labelled EVs (error bars represent 95% confidence interval of the mean) in diluent C (n = 7, *p* value <0.05).

Further confirmation of PKH induced larger species was done by varying the concentration of EVs while holding the PKH concentration constant. The PKH concentration (0.16 µM) used was the level shown to generate fluorescently detectable PKH-labelled EVs (Fig. 2B). Representative examples of the particles’ size distribution measured by NTA for different concentration of EVs labelled with PKH dye molecules can be seen in (Fig. 4A–D). Additionally, quantitative determination of NTA results was conducted by comparing the size modes of the nanoparticles (Fig. 4E). As expected, generation of larger species was observed by size distribution as well as the shift in the size mode regardless of the EVs concentration tested.

**Figure 5:**
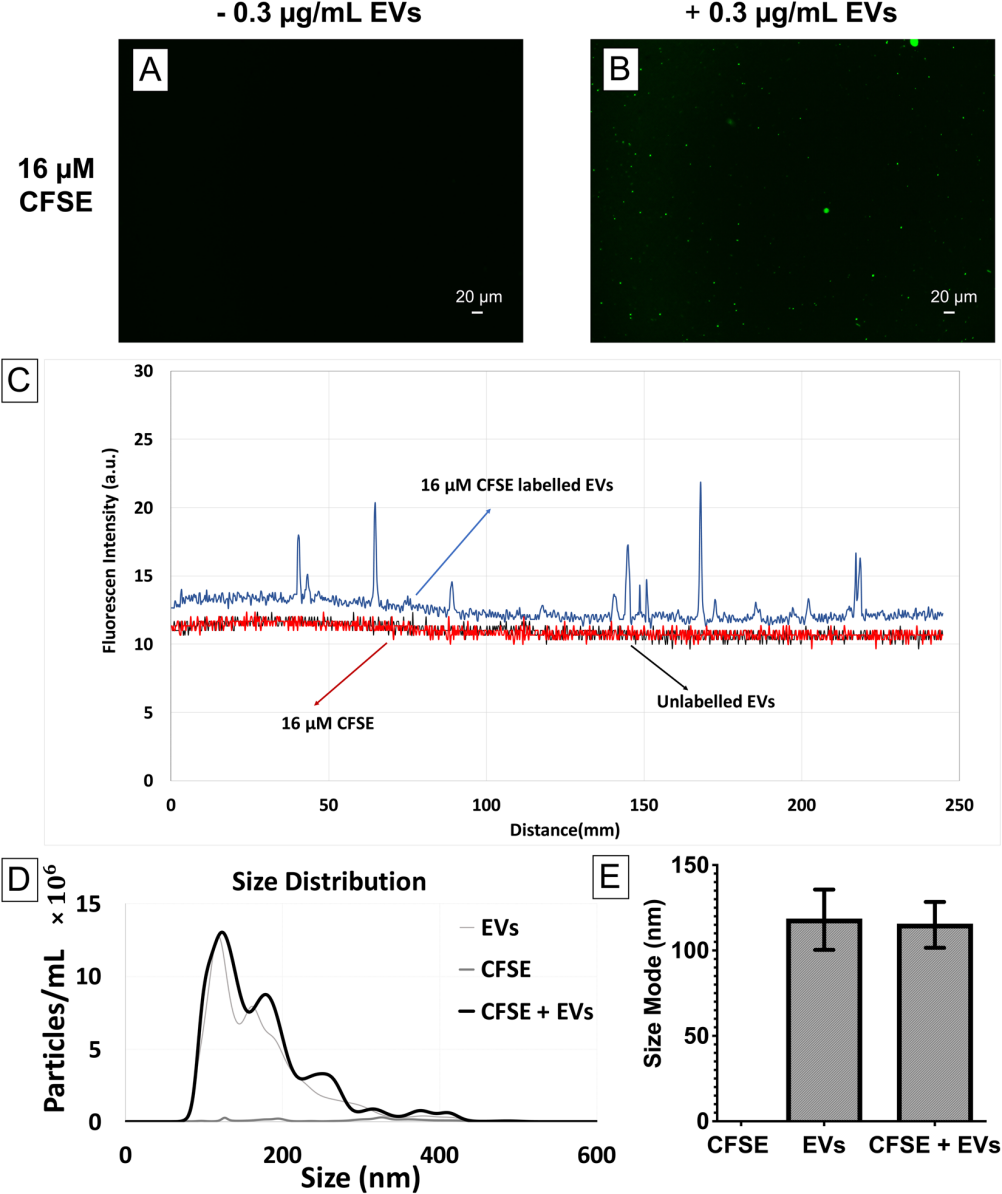
Size characterization of CFSE labelling of EVs by Nanoparticle Tracking Analysis (NTA). Fluorescent images of (**A**) 16 4 µM CFSE only control (**B**) 4 µM CFSE-labelled EVs (0.3 µg/mL) in PBS. (**C**) Representative line scan analysis of fluorescent images (**A,B**). (**D**) Size distribution of EVs only control, CFSE only control, and CFSE-labelled EVs in PBS (n = 7). (**E**) Size mode of EVs only control, CFSE only control, and CFSE-labelled EVs (error bars represent 95% confidence interval of the mean) in PBS n = 7, *p* value < 0.05).

As opposed to lipophilic dye molecules which may cause a size shift towards larger particles, potentially through PKH nanoparticles fusion/aggregation or PKH dye molecules intercalation with EVs, it is anticipated that direct luminal labelling of EVs with fluorescent compounds will not change the size of EVs. In order to study this hypothesis, EVs were labelled with the CFSE luminal binding dye using a previously established protocol^34^. Compared to the EVs only control and CFSE dye control (Fig. 5A), several brighter features were found in their fluorescent images (CFSE dye + EVs) which are likely the CFSE-labelled EVs (Fig. 5B). Additionally, the line scan obtained from the CFSE-labelled EVs showed intensity spikes indicating the presence of CFSE-labelled EVs (Fig. 5C). In contrast to PKH dye, NTA analysis showed that CFSE did not form nanoparticles in the CFSE only control (Fig. 5A,D), which was in agreement with the findings of Morales-Kastresana *et al*.^34^. Size distribution and quantitative determination of the particle size modes measured by NTA showed no significant change in CFSE-labelled EVs compared to unlabelled EVs (Fig. 5D,E). This result suggests that luminal labelling by luminal binding dye (CFSE) preserve the size of EVs after labelling which makes this type of dye more reliable when compared to PKH.

## Discussion

All cell types are capable of shedding membrane-enclosed vesicles called EVs that play a key role in intercellular communication. EVs have been found in diverse bodily fluids including blood, urine, saliva, amniotic fluids, ascites, cerebrospinal fluid and breast milk^2,35^. The use of extracellular vesicles for diagnostic and therapeutic applications has seen a major interest increase in recent years because of their capability to exchange components such as nucleic acids, lipids and proteins between cells^36^.

To investigate the fate of EVs, many imaging tools and labelling methods have been developed. Electron and optical microscopy are the most commonly used techniques for visualizing EVs^37^. However, due to the small size of EVs, their tracking can be challenging and they need to be fluorescently labelled in their native state. Lipophilic labelling with dye molecules such as the PKH family have been widely used to label a range of cell types such as mesenchymal stem cells^38,39^ and tumor cells^40^ in proliferation and migration studies^39,41^. Since EVs have a lipid bilayer structure similar to that of the cell’s plasma membrane, the PKH family has been adapted for EV labelling due to the hydrophobic interactions between the EV lipids and the long alkane tails of PKH. Internalization of PKH-labelled EVs by dendric cells^9^, macrophages^17^, endothelial cells^8^ and fibroblasts^8^ has been reported.

Using polymeric, organic and inorganic nanoparticles, it has been shown that size, shape, surface chemistry and hydrophobicity of nanoparticles are important factors in their uptake and biodistribution^19–22^. The effect of nanoparticle size alone on their uptake has been investigated using different types of nanoparticles and cells such as mesoporous silica nanoparticles on HeLa cells by Lu *et al*.^28^, fluorescent latex beads on B16 cells by Rejman *et al*.^42^, as well as polystyrene nanoparticles on Caco-2 and MDCK cells by Kulkarni *et al*.^23^. In addition to cellular uptake efficiency, Rejman *et al*.^42^ have shown that nanoparticles smaller than 200 nm can be taken up by clathrin-coated pits, while larger particles tend to be internalized by caveolae-mediated processes. Moreover, Kulkarni *et al*.^23^ studied the biodistribution of intravenously injected polystyrene nanoparticles in different organs of rats. The authors found 200 nm nanoparticles showed higher accumulation in both liver and spleen compared to 100 nm nanoparticles. All taken together, these evidences suggest a size shift from 100 nm to 200 nm decreases the cellular uptake efficiency and kinetics and affects their biodistribution. Although studies have recognized the importance of size of nanoparticles in uptake and biodistribution, the research has yet to systematically investigate the effect of fluorescent labelling on the size of EVs. Importantly, fluorescent labelling must preserve the size of EVs since any size change may alter the uptake and distribution of EVs.

The size and concentration of nanoparticles including EVs have been determined using different techniques with their own advantages and limitations such as electron microscopy (SEM and TEM), atomic force microscopy (AFM), dynamic electron microscopy (DLS) and nanoparticle tracking analysis (NTA). NTA is a widely used technique in the field and is of particular interest in this study since it is quick to perform and it provides a detailed analysis of the measuring sample leading to statistically significant nanoparticles’ size distribution. Furthermore, NTA does not require processing procedures such as drying and coating which can affect the nanoparticles properties^43–46^. Hence, the present study set out with the aim of assessing the impact of PKH labelling on the size of EVs using NTA. The concentration of both PKH dye and EVs were systematically varied and the particles’ size distribution was determined by NTA. In all conditions tested, NTA analysis revealed a size mode shift from ~100 nm for unlabeled EVs to ~200 nm for PKH labelled EVs.

In agreement with our findings, Salatin *et al*. used the lipophilic tracer dialkylcarbocyanine (DiI) to enhance clustering and aggregation of EVs which was confirmed by electron microscopy and flow cytometry^47^. Furthermore, Pospichalova *et al*. found that another lipophilic dye, styryl dye (also referred to as FM dye), results in larger EVs after labelling which was characterized by flow cytometry^48^. Additionally, Morales *et al*. studied different types of labelling methods for nanoscale flow cytometry. Morales *et al*. main focus was to identify the method that generates fewer background contaminants during the labelling process and not the effect of labelling on EVs size. Their NTA data also showed a size shift towards larger particles after PKH labelling of EVs, however this was not mentioned by the authors in the study^34^. Gangadaran *et al*.^28^, examined the biodistribution of EVs using DiR (a similar lipophilic dye as PKH) and found that lipophilic labelling of EVs increased the localization of EVs in liver and spleen. This change in the biodistribution may be due to the increase in size of EVs after labelling by lipophilic dye molecules. These previously reported results further support our findings regarding the size change of EVs after labelling with lipophilic dye molecules such as the one belonging to the PKH family. In summary, the size shift towards larger particles caused by PKH labelling of EVs is likely to change the cellular uptake level and internalization mechanism as well as the biodistribution of EVs, reducing its validity as an EVs tracer.

In addition to the concern regarding the size change of EVs after labelling, recent studies have highlighted generation of artifacts such as formation of numerous nanoparticles which consist exclusively of micelles/aggregates of PKH, without EV content^33,34,49^. It was further shown that in terms of size, surface area and fluorescent intensity, the PKH nanoparticles cannot be distinguished from PKH labelled EVs and were taken up by astrocytes. Therefore, this capacity for cell uptake of PKH nanoparticles may lead to false positive signals in EV tracking studies^33^. However, cyanine-based membrane probes called MemBright have been recently developed which do not form nanoparticles which is essential for tracking EVs, in contrast to the commonly used PKH family^50,51^.

One alternative to lipophilic dye molecules that stain the membrane of EVs, is luminal labelling such as CFDA-SE. CFDA-SE dye molecules are membrane permeable chemical compounds that covalently bind to primary amine inside EVs and fluoresce after ester hydrolysis of the dye in the lumen of the EVs which forms active CFSE molecules^52–55^. Therefore, we hypothesized that luminal binding dye molecules do not affect the size of EVs. In order to study this hypothesis, the luminal binding dye CFSE was used to label EVs and the size of EVs was determined before and after labelling with NTA. NTA results showed that as opposed to PKH labelling of EVs which increased the size of EVs, CFSE dye labelling maintained the normal size of EVs which precludes any size related cellular uptake and biodistribution aberrancies. Consistent with our finding, Morales-Kastresana *et al*.^34^ and Pospichalova *et al*.^48^ found luminal binding fluorescent compounds did not form nanoparticles and did not increase EVs size after labelling using flow cytometry.

Here, NTA was employed to systematically explore the effect of PKH labelling on the size of EVs by changing the PKH to EVs ratio. In all conditions tested, a size mode shift towards larger particles was observed after PKH labelling of EVs which may cause aberrancies in cellular uptake, biodistribution and half-life circulation. This observed size shift combined with other previously reported artifacts such as formation of PKH nanoparticles suggest that the PKH family dye is not reliable for labelling EVs. In contrast to the lipophilic class such as PKH, luminal binding dye molecules like CFSE did not cause a size shift in labelled EVs, suggesting that CFSE may be a better labelling option for EVs by preserving the size of EVs after labelling. However, It is important to note that the effect of CFSE labelling of EVs on their biological behavior such as their uptake and biodistribution needs to be further explored. Furthermore, CFSE is not the only alternative for lipophilic dye molecules such as PKH and suitability of other fluorescent labelling methods needs to be investigated in future studies using different techniques such as NTA, flow cytometry and electron microscopy.

## Materials and Methods

### EVs labelling with lipophilic dye (PKH)

Lyophilized urinary CD63, CD9, CD81 positive EVs (HansaBioMed, Estonia) were diluted in ultra-pure water to a protein concentration of 100 µg/mL, following manufacturer instructions. Prior to staining, 1.2 µL of 1 mM PKH26 stock (Red Fluorescent Cell linker for General Cell Membrane, Sigma-Aldrich) was added to 300 µL of diluent C and incubated at 37 °C for 15 minutes. Then, 1 µL of EVs stock was added to PKH26 in diluent C, resulting in a sample with final concentrations of 0.3 µg/mL of EVs and 4 µM of PKH26. Other concentrations of EVs (4.5, 1.5 and 0.03 µg/mL) and PKH (20 and 0.16 µM) were made following the same overall procedure in diluent C.

### EVs labelling with luminal binding dye (CFSE)

5-(and-6)-Carboxyfluorescein Diacetate Succinimidyl Ester (CFSE) stock was made following the manufacturer instructions (CellTrace CFSE Cell Proliferation Kit, Thermo Scientific Fisher) by adding 18 µL of dimethyl sulfoxide (DMSO, Sigma-Aldrich) to the CFSE dye resulting in a 5 mM stock. In order to stain EVs with CFSE, 1 µL of CFSE stock was added to 300 µL of PBS prior to staining and then 1 µL of EVs from 0.1 µg/mL EVs stock was added and incubated for 2 hours at 37 °C, as was previously described^34^. In order to remove unbound dye molecules, the samples were ultracentrifuged (Optima MAX-XP, Beckman Coulter) at 100000 × g for 60 minutes at 4 °C (TLA 110 rotor with cleaning factor of 81).

### Nanoparticle tracking analysis (NTA)

For each run, 300 µL of the prepared samples were injected into the sample chamber of a NS300 instrument (NanoSight, Aumesbery, UK) with a 532 nm green laser. Seven measurements of each sample were performed for 30 seconds each. For the “Blur”, “Minimum expected particle size”, and “Minimal track lengths” the auto adjustment settings provided by software developer were used. The camera level (9–12) and detection threshold (2–6) were adjusted manually for each experiment as recommended by the manufacturer. For data capturing and analysis, the NTA analytical software (NanoSight NTA version 3.2) was used. Briefly, from the recorded video, the mean square displacement of each detected particle was determined. Then, using the Stokes-Einstein equation, the diffusion coefficient and sphere-equivalent hydrodynamic radius were determined by the software.

### Statistical analysis

The size mode quantitative analysis is presented as means with error bars represent 95% confidence interval of the means. The comparison between the size mode of EVs before and after labelling were carried out using the two-tailed student *t* test. Differences were considered significant at *p* < 0.05.

### Fluorescent imaging and analysis

Fluorescent images were taken using a Keyence BZ-X700 microscope (Keyence Corp. of America, MA, USA) with the same exposure time for all samples. The line scan analysis was conducted using the NIH ImageJ software version 1.52.
